# High levels of healthcare utilization prior to diagnosis in idiopathic pulmonary arterial hypertension support the feasibility of an early diagnosis algorithm: the SPHInX project

**DOI:** 10.1177/2045894018798613

**Published:** 2018-09-06

**Authors:** Rito Bergemann, James Allsopp, Harvey Jenner, Flora Ashley Daniels, Edmund Drage, Yevgeniy Samyshkin, Claude Schmitt, Steven Wood, David G. Kiely, Allan Lawrie

**Affiliations:** 1 GlaxoSmithKline, Brentford, Middlesex, UK; 2 Current affiliation: Evalueserve Life Sciences Healthcare, London, UK; 3 Scientific Computing Group, Sheffield Teaching Hospitals Foundation Trust, Sheffield, UK; 4 IQVIA, London, UK; 5 Department of Infection, Immunity & Cardiovascular Disease, University of Sheffield, Sheffield, UK; 6 National Pulmonary Hypertension Service (Sheffield), Sheffield Pulmonary Vascular Unit, Royal Hallamshire Hospital, Sheffield, UK; 7 INSIGNEO, University of Sheffield, Sheffield, UK

**Keywords:** idiopathic pulmonary arterial hypertension, right heart catheterization, diagnosis, real-world data, Hospital Episode Statistics

## Abstract

Idiopathic pulmonary arterial hypertension (iPAH) is a rare progressive, life-shortening disease, usually diagnosed at an advanced stage. We hypothesize that patients with iPAH exhibit patterns of health-seeking behavior before diagnosis that will allow the development of earlier identification tools. The Sheffield Pulmonary Hypertension IndeX (SPHInX) project aims to develop a predictive algorithm based on routinely collected healthcare resource utilization (HCRU) data. This report focuses on the initial feasibility of the project, examining whether Hospital Episode Statistics (HES) data from the National Health Service in England have sufficient richness to support the development of an early diagnosis algorithm. This is a two-stage study. First, hospital interactions during 2009–2014 captured in HES data identified 127,815 adult patients with pulmonary hypertension (PH) ICD-10 codes, containing a probable iPAH cohort with incidence and demographics similar to the reported literature. HCRU was high in the three years before diagnosis. Second, to examine HCRU in patients with a confirmed iPAH diagnosis, we built the SPHInX dataset incorporating all patients investigated for suspected PH in the Sheffield Pulmonary Vascular Disease Unit during 2008–2016 (n = 6674). For the SPHInX dataset, data could be linked to HES in 98.6% of cases and patients with confirmed iPAH had similar levels of pre-diagnosis HCRU. In conclusion, patients with probable iPAH identified using HES and patients with confirmed iPAH have high levels of HCRU for several years before diagnosis. Artificial intelligence models will now be used to develop the SPHInX algorithm to screen for undiagnosed iPAH in the general population.

Pulmonary arterial hypertension (PAH) is a rare, progressive, and life-shortening condition that, untreated, leads to right ventricular failure and death.^1^ It may be associated with an underlying cause, but is often idiopathic (iPAH).^2,3^ The prevalence and incidence of iPAH varies by country and region and is reported to be 5–20 per million and 1.0–3.3 cases per million population per year, respectively.^3–5^ At the time of diagnosis, iPAH is frequently advanced, and untreated the median survival is <3 years. While there is no cure for iPAH, treatments have evolved over the past 10 years, resulting in improvements in symptoms, exercise capacity, hemodynamics, time to clinical worsening, and/or survival.^6^

Symptoms of iPAH, such as breathlessness and fatigue, are non-specific and the clinical signs are subtle until disease is advanced.^7^ Diagnosis is usually first suggested by echocardiography and confirmed by right heart catheterization (RHC).^6^ Despite emerging evidence that early treatment is associated with better outcomes,^8–10^ the typical delay between the onset of symptoms to diagnosis of iPAH of 2–3 years has not improved in the past 20 years.^11,12^ Registry data confirm that at the time of diagnosis, iPAH is usually advanced from a hemodynamic perspective.^13^ In patients with systemic sclerosis, the prevalence of PAH is high (∼10%)^14^ and screening programs^13,15–17^ have been shown to improve diagnostic rates and permit early detection of patients with less severe hemodynamic disease. While screening is available in patients with systemic sclerosis to identify PAH earlier due to its high prevalence,^17–19^ no such approach is currently available for iPAH.

The goal of the overall Sheffield Pulmonary Hypertension IndeX (SPHInX) project is to develop a novel screening algorithm to identify patients with iPAH at an early stage of their disease. To date, no group has systematically looked at healthcare activity before diagnosis to support early diagnosis of iPAH. We hypothesized that existing patient characteristics, which can be accessed from coded entries in national healthcare databases for hospital activity, have the potential to facilitate early diagnosis of patients with iPAH. To facilitate this, the first phase of this project and the focus of this manuscript was to determine whether routinely collected data obtained from the National Health Service in England, captured in the Hospital Episode Statistics (HES) dataset, has the potential to support the development of a predictive algorithm for iPAH. In addition, we also describe the development of the SPHInX project.

## Methods

### Phase 1 – pilot study: exploring HES data to detect patients with iPAH and assess their healthcare resource use prior to diagnosis

**Fig. 1.**
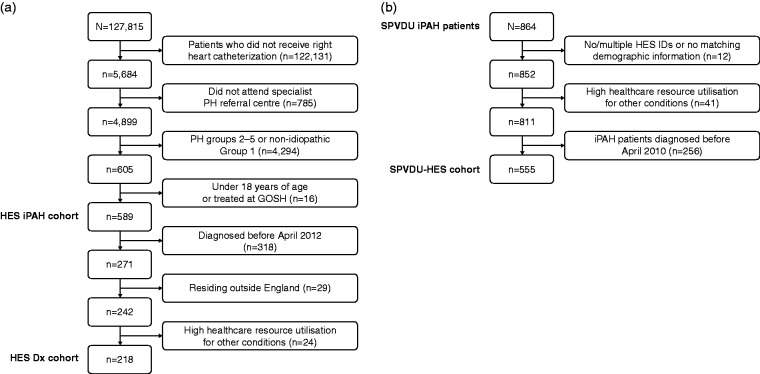
Patient flow in the pilot study. Dx, diagnosis; GOSH, Great Ormond Street Hospital; HES, Hospital Episodes Statistics; PH, pulmonary hypertension; iPAH, idiopathic pulmonary arterial hypertension; SPVDU, Sheffield Pulmonary Vascular Disease Unit.

Patients were identified from HES data obtained from NHSE between April 2009 and October 2014. Figure 1a shows the process used to select patients with iPAH. International Statistical Classification of Diseases and Related Health Problems, version 10 (ICD-10) codes for pulmonary hypertension (PH) (I27.0, I27.2, I27.9) were used to identify potential patients with iPAH. Patients were excluded if they: (1) did not undergo RHC; (2) did not attend a specialist PH referral center; (3) had an ICD-10 code for non-iPAH (Supplementary Table 1); (4) were aged < 18 years or had attended the UK specialist PH center for children at Great Ormond Street Hospital, to yield the HES probable iPAH cohort. The annual incidence of new probable iPAH diagnoses for each complete year of data for 2010, 2011, 2012, and 2013, and the age and gender distributions for the complete dataset, were determined. Annual incidence and age and gender distribution of our probable iPAH cohort were compared to data available from the published literature from the UK, France, and Switzerland.^3,5,16,20^

Following examination of the data within the HES probable iPAH cohort, patients were further excluded if they: (5) were diagnosed before April 2012 with < 3 years of HES data available pre-diagnosis in the dataset (defined by date of RHC at one of the six specialist PH centers); (6) resided outside England; or (7) had high healthcare resource utilization (HCRU) ≥ 250 HES events, to ensure that within this HES probable iPAH cohort, each patient was suitable for the planned analysis. The mean number of hospital events, as well as the frequency and type of specialties visited across all care settings (accident and emergency [A&E], inpatient, and outpatient) in the three years (Y3), two years (Y2), and one year (Y1) before diagnosis with iPAH were calculated.

### Phase 2: linking HES to Sheffield datasets to build the SPHInX cohort

The Sheffield Pulmonary Vascular Disease Unit (SPVDU) and the University of Sheffield have one of the largest iPAH-enriched diagnostic clinical datasets in the world. Data from three Sheffield databases (ASPIRE Registry,^13^ InfoFlex database containing operational clinical data including diagnostics and follow-up investigations, and the ArQ [Sheffield PH Biobank] database) were merged with data included from December 2008 to October 2016 for 6674 unique patients evaluated for suspected PH during this period. Patients were linked to the HES dataset via their NHS number to form the SPHInX database and patients with a confirmed iPAH diagnosis selected. This allowed us to build a dataset comprising novel sources of real-world data (national HES data, clinical data for all PAH patients with suspected PH managed by Sheffield Teaching Hospitals NHS Foundation Trust [STHFT], and University of Sheffield/STHFT PH biobank data), subtype patients who attend the Sheffield center using clinical experts’ validated data, and confirm PAH diagnoses and subtype. This would not be possible with HES data alone due to the heterogeneous use of ICD-10 codes for iPAH. Patient demographic characteristics (e.g. gender, month and year of birth, and general practitioner postcode) were assessed to ensure consistent linkage of patient data between the SPHInX database and HES data.

### Validation of first stage study findings using confirmed iPAH diagnosis from the SPHInX database

Patients with confirmed iPAH and HES data collected between April 2000 and March 2017 (inpatient care: April 2000–March 2017; outpatient care: April 2003–March 2017; A&E: April 2007–March 2017) were identified from the SPHInX database. Patients were excluded if: (1) there was no HES identifier or demographic data did not match between the SPHInX dataset and HES data; (2) they had high HCRU with ≥ 250 HES events; or (3) < 3 years of data before the index event were available (i.e. patients diagnosed before April 2010) (Fig. 1b). From this linked SPHInX cohort, we determined the: (1) index date, defined as the last relevant (cardiology, respiratory medicine, or neurology specialist event) in their record before their first visit to the SPVDU (this is, on average, 76 days before their first visit); (2) lookback date, defined as the earliest date of the patient’s first event captured in HES, or the event occurring five years before the patient’s index date; (3) first iPAH symptom, defined as the first HES event assigned one of 141 ICD-10 and Classification of Interventions and Procedures (OPCS) codes in the primary diagnosis field, derived from a blended data and clinically driven approach for symptoms and procedures aligned to iPAH patients; and (4) patient’s first Sheffield visit, based on the date of the patient’s first visit captured in the SPHInX datasets.

For patients with iPAH identified from the SPHInX database, the mean number of hospital events, as well as the frequency and type of specialties visited across all care settings (A&E, inpatient, and outpatient), in years 1, 2, and 3 between lookback and index dates were calculated.

### Ethical approval

An overview of the interdependency of data and details of the ethical approvals required at each stage of the analyses are shown in Supplementary Figure 1. Relevant permissions and approvals were sought and obtained from the East Midlands – Derby Research Ethics Committee (ref: 16/EM/0286), and Confidentiality Advisory Group (CAG), for the linkage of datasets under Section 251 of the Health and Social Care act 2014 (ref. no. 16CAG0091). The Independent Group Advising on the Release of Data (IGARD) at NHS Digital approved the use of HES data for this study. The process to receive these permissions required research approvals from the STHFT Caldicott Guardian. We also sought and received a letter of support for the research from the Pulmonary Hypertension Association UK (PHA UK) patient advocacy group. Any patient who had opted out of research was removed from our analyses.

## Results

### Phase 1: identification of iPAH using HES data from NHSE (pilot study)

**Fig. 2.**
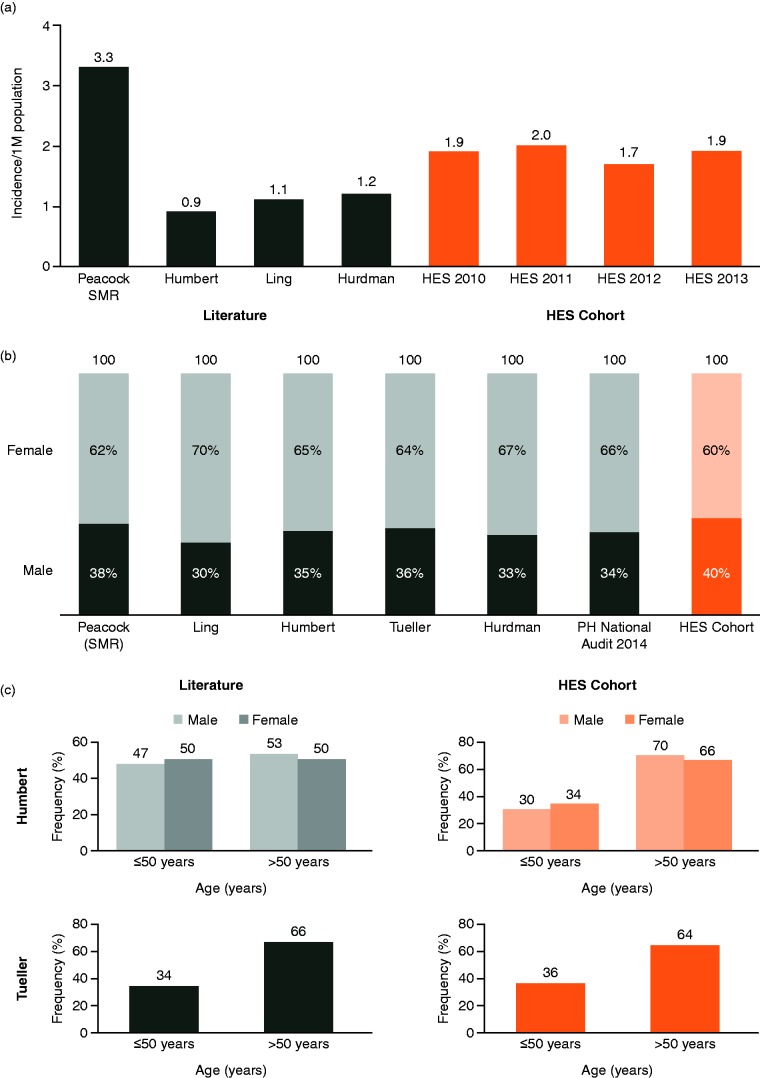
Comparison of demographics of HES probable iPAH cohort vs. reports from literature for (a) incidence per million population, (b) gender distribution, and (c) age. FR, France; HES, Hospital Episodes Statistics; M, million; SMR, Scottish Morbidity Record; SPVU, Scottish Pulmonary Vascular Unit.

Data extracted from HES between 2009 and 2014 identified 127,815 patients with an ICD-10 code for PH (I27.0, I27.2, I27.9). Application of the selection criteria identified a cohort of 589 patients with probable iPAH (Fig. 1a). This corresponds to an incidence of 1.9/million in 2010, 2.0/million in 2011, 1.7/million in 2012, and 1.9/million in 2013 (Fig. 2a). The incidence of probable iPAH diagnosed from HES was within the range of reported incidences in the literature, which was in the range of 0.9–3.3/million per year. Similarly, for gender distribution, 60% of patients in the HES dataset were women, compared to the published literature range of 62–70% (Fig. 2b). The age distribution of patients identified from HES data was higher compared with historical studies, with 64% aged > 50 years, although this was similar to more recent series (Fig. 2c).

### Phase 1: hospital-related activity identified from HES before diagnosis of iPAH (pilot study)

**Fig. 3.**
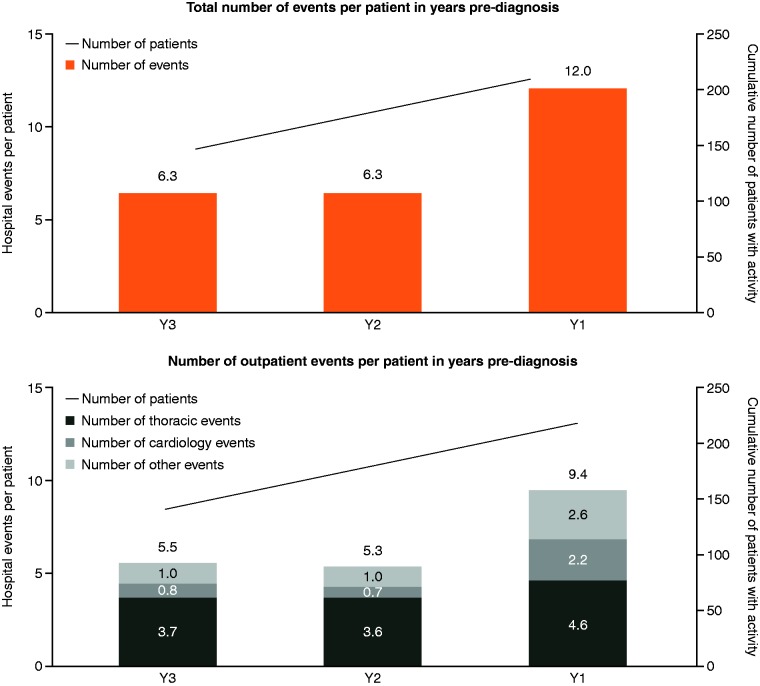
HES diagnosis cohort patients have a high mean number of hospital events in the years preceding diagnosis. Dx, diagnosis; Y, years preceding diagnosis.

Data for 218 patients with probable iPAH with at least three years of data before diagnosis (April 2012 to October 2014) (Fig. 1a) were analyzed and the mean number of hospital events, as well as the frequency and type of specialties visited across all care settings (A&E, inpatient, and outpatient), in years 1, 2, and 3 before diagnosis are shown in Fig. 3. In the three years before diagnosis, the average patient had approximately 25 hospital events; 12 events were within one year of RHC, of which six were with a cardiologist or respiratory clinician. Most hospital events were outpatient events, with an average of 20.2 per patient in the three years before diagnosis, with 9.4 of these occurring in the year before diagnosis; cardiology or thoracic events accounted for approximately half (4.8 events in total). NHSE contact within the three years before diagnosis encompassed an average of 2.4 hospital trusts and six different specialties (data not shown).

### Phase 2: building the SPHInX cohort with HES

The SPHInX dataset, which merged data from three separate databases, identified 6674 patients who were investigated for suspected PH between 2008 and 2016, detecting 864 patients with confirmed iPAH, 3944 patients with other forms of PH, and 1383 patients with no PH (483 patients had no assigned final diagnosis) and linked to HES data. For patients with confirmed iPAH, we were able to link HES data for 852 out of 864 of these patients, representing 98.6% of all IPAH patients diagnosed with IPAH in Sheffield in this period of time.

### Hospital-related activity identified from the SPHInX database before index event (validation study)

**Fig. 4.**
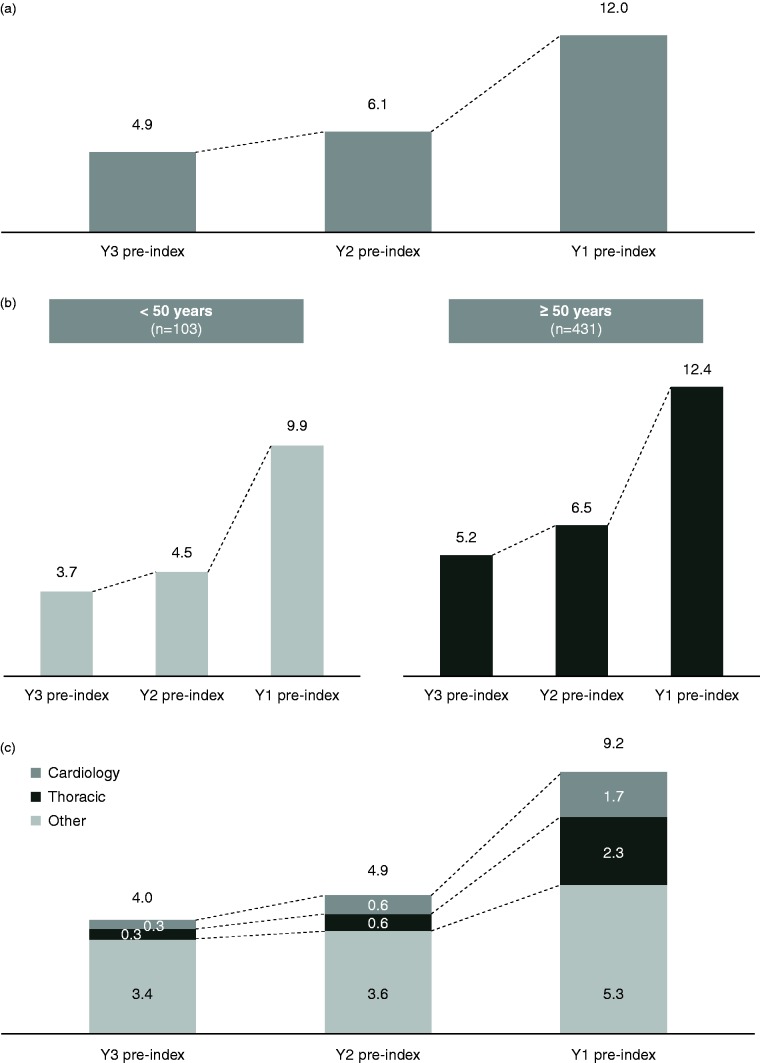
An increased usage of secondary care in the year preceding diagnosis within the SPVDU-HES cohort.

Within the overall confirmed iPAH cohort, 85% of patients had recorded HES events dating back five years before the index date. Among patients with confirmed iPAH, their first symptom was recorded in General Medicine (36.0%), Respiratory Medicine (32.8%), Cardiology (13.3%), Geriatric Medicine (3.1%), Accident & Emergency (2.9%), and General Surgery (1.1%). Data for 555 patients of the SPHInX iPAH cohort having at least three years of data before diagnosis (April 2010 to March 2017) were analyzed and the mean number of hospital events, as well as the frequency and type of specialties visited across all care settings (A&E, inpatient, and outpatient), in years 1, 2, and 3 before index date is shown in Fig. 4. In the three years before index date, the average patient had approximately 23 hospital events (Fig. 4a). Patients aged ≥ 50 years had more hospital events in the three years before the index date than patients aged < 50 years (Fig. 4b). Most hospital events were outpatient events, with an average of 18.1 per patient in the three years before the index date; cardiology or thoracic events accounted for approximately 32.0%, with an average of 5.8 total events during the three years before the index date (Fig. 4c).

## Discussion

In this study, we have demonstrated that patients with probable iPAH can be identified from national databases using ICD-10 codes with diagnostic rates similar to those reported in various registries. Patients with probable iPAH identified using this approach demonstrate high levels of HCRU before diagnosis. We subsequently confirmed these findings in a carefully characterized cohort of patients with confirmed iPAH from the SPHInX database, and in doing so demonstrate that patient level hospital data can be linked to national HES data with high rates of success (98.6%). The data available from routinely collected HES have a richness and complexity that may potentially facilitate early diagnosis by identifying a fingerprint to detect patients at risk of iPAH. A model based on artificial intelligence techniques will now be used to develop the SPHInX algorithm; and if this demonstrates sufficient diagnostic accuracy, we plan to deploy it in the general healthcare user population to screen for patients with undiagnosed iPAH.

The HES dataset has full population coverage of activity for all patients accessing NHSE care, across outpatient, inpatient, and A&E settings, and allows for descriptive analysis of patients. However, the dataset has several limitations, including no specific ICD-10 code for iPAH, with the code “primary pulmonary hypertension” often used to diagnose patients with PAH, making it challenging to explore HCRU in iPAH patients without the use of techniques to further validate this cohort, multiple different pulmonary diseases being coded under the same ICD-10 code, the absence of prescription data, and the collection of less detail for patients entered in the A&E and outpatient settings, compared with the inpatient setting. Nonetheless, we consider the HES dataset used to identify patients with iPAH to be robust based on the selected cohort and to correlate well with the characteristics of patients with iPAH described in the medical literature from the UK, France, and Switzerland;^3,5,16,20^, however, we acknowledge that it is likely to underdiagnose patients with iPAH and, indeed, the annual incidence of iPAH from the SPHInX database was several-fold higher. Nonetheless, it is interesting that our iPAH cohort identified using HES data was very similar demographically to those patients with a confirmed diagnosis of iPAH from the SPHInX database who had similar levels of activity pre-diagnosis (i.e. 17.7 events in the three years before diagnosis and a similar proportion of patients seeing respiratory and cardiology specialists). This highlights the opportunity to interrogate activity data from HES to see if a fingerprint can be identified, based on frequency, type and temporal relationship of activities, that may detect patients at risk of iPAH. While we believe the opportunity here is considerable, the barriers to constructing SPHINX have been substantial and may well have meant that there are other conditions with similar characteristics to iPAH where this approach could be beneficial but is unlikely to be undertaken. In particular, the duration of time (17+ months) to secure the relevant permissions for the varies bodies and obtain the required data is a substantial challenge in pursuing this type or research.

Screening is advocated for several conditions to facilitate early diagnosis. However, while screening can improve patient outcomes, it may also be detrimental; several factors must be carefully considered when contemplating screening initiatives to safeguard patients. Screening must have the likelihood of improving long-term outcomes for patients; it must have a minimal risk of falsely identifying healthy individuals or over diagnosing the disease. The economic impact of the screening and any subsequent interventions must also be carefully weighed. Currently within NHSE, a number of screening programs exist to facilitate early diagnosis of cancer and to identify patients at risk of cardiovascular events, where early treatment may result in cure or strategies may reduce the development of vascular complications.^21^ PAH is a challenging condition to diagnose due to the non-specificity of symptoms and the invasive nature of tests required to confirm its presence. Consequently, patients tend to present when the disease is advanced. The population can be enriched for patients at risk of developing PAH, such as in systemic sclerosis where screening is advocated in asymptomatic patients due to its high prevalence (10%), or in symptomatic patients with portal hypertension or human immunodeficiency virus infection, where the prevalence is lower compared with systemic sclerosis (but still significantly higher versus the general population). Strategies to try and diagnose patients with iPAH at an earlier stage of disease have focused on increasing disease awareness and a systematic approach to the investigation of the breathless patient, in the hope that this will facilitate improved diagnostic rates. The results for iPAH, however, remain very disappointing with the efforts of the last 20 years achieving no reduction in time from initial iPAH symptom to diagnosis, and with the majority of patients still presenting with advanced disease in World Health Organization functional class III and IV. The emergence of large and complex datasets describing healthcare behavior, and the development of approaches to allow them to be analyzed using machine-learning techniques, provide us with an opportunity to explore novel methods to identify patients with iPAH.

This study found high levels of activity and frequent contacts with healthcare services in the three years before a confirmed diagnosis of iPAH. In addition, patients frequently attended respiratory and cardiology specialties. The combination of high levels of activity and the richness of specialty interactions raises the possibility that patients with iPAH may exhibit characteristic patterns of behavior separate from other cardiorespiratory conditions, providing sufficient data to support the development of a predictive diagnostic algorithm for iPAH.

We are currently engaged in a big-data approach, utilizing a model based on artificial intelligence to develop a predictive algorithm to screen for patients with undiagnosed iPAH in the general population.
